# Supplementary material for: Musculoskeletal misdiagnoses in children with brain tumors: A nationwide, multicenter case-control study
Source: PLoS One. 2023 Jun 23;18(6):e0279549. doi: 10.1371/journal.pone.0279549 (PMC10289381; doi:10.1371/journal.pone.0279549)
Supplement: Attachment — Submitted filename: response to reviewer.docx [file pone.0279549.s003.docx]

We hereby submit our revised manuscript PONE-D-22-33822 “Musculoskeletal misdiagnoses in children with brain tumors A nationwide, multicenter case-control study”.

We would like to thank you and the reviewers for your time, the careful examination, and comments of the manuscript. Below all the comments addressed has been responded separately. Point-by-point responses are written in *italic*. Changes in the manuscript are written in red text-typ in the “Main document – marked Copy”. Please find the details below.

**Response to comments from reviewers**
Reviewer 1:

Reviewer #1: General comments:
This study examined whether prior musculoskeletal diagnoses in childhood brain cancer are misdiagnosed and affect diagnostic delay. The topic and nationwide medical chart data are interesting. However, the major limitations of this study include a small sample size, which cannot draw a firm conclusion for observed non-statistical differences. Below are comments about this manuscript.

Major comments:
#1. How did the authors manipulate the year of diagnoses in the matching process? Because the study period extended 21 years from 1996 to 2018, the diagnostic procedures/modalities and treatments must have advanced. If comparing cases in the earlier cohort to later controls without considering diagnostic periods, confounding of those medical advances may arise.

*Response: Thank you for your considerations, which are appreciated. You are right about the treatment and procedure changing over time. The cases and controls were equally distributed over time. We added the following to the results, page 7: The prevalence of the cases and controls did not differ when dividing into the two periods, as 64% of the cases and 61% of the controls were diagnosed in the period 1996-2006 (p = 0.75) and 39% cases and 36% controls were diagnosed from 2007-2018.*

#2. The discussion and conclusion sections, including the abstract and main text, should be cautious about concluding a strong interpretation, such as “musculoskeletal misdiagnoses in childhood brain tumors do not affect the diagnostic delay.” One primary reason for this non-significant result is the weak statistical power. In fact, in Fig 3 and Fig 4, the results suggest that cases tended to have better survival and total interval outcomes. For safer to say that this study did not find a significant association. With insufficient statistical power, please be advised to tone down such decisive conclusions/messages.

*Response: The conclusions have been toned down in the abstract, discussion and conclusion*

Minor comments:
#1.Page 6, Result section, the first paragraph: “From January 1996 … 46% (39/84) were girls.” This whole paragraph may be moved to the Method section (probably, in between the Data collection and Statistical analysis parts), with some subheadings such as “Cases and controls.” Because in the Method section, details for the matching process should be described, which helps readers understand the design easily.

*Response: The section has been moved, and a new introduction to the result section has been added*

Reviewer #2: Thank you to the editor for the opportunity to review this interesting manuscript entitled “Musculoskeletal misdiagnoses in children with brain tumors: A nationwide, multicenter case-control study”

It is important to get so called negative findings to published, too.

Regarding abstract: I would suggest to add to results the finding of “7-fold risk of musculoskeletal symptoms in infratentorial tumors”. This is given on page 13.

*Response*: *A sentence of this result has been added to the abstract*

In introduction, second row:… overall survival rate has shown…”. Please add survival rate of brain tumors in Denmark. This because you only refer to the Danish data (ref 3).

*Response: We have added this.*

On row 5 about diagnostic delay you may consider to add a recent reference from Swedish register data (Rask o et al, Pediatr Blood Cancer. 2022 Nov;69(11):e29850)

*Response: the reference has been added*

About study designs, could you please provide reasoning for choosing only 2 control patients per each case as you, however, had more than 900 patients with BT-diagnosis.

*Response: We choose to include two controls per case as we hereby were able to secure controls being both age- and gender-matched and diagnosed with CNS tumors in the same period of time (+/- one year). Further, we do not believe that a higher number of controls would have changed the essence of the results.*

In discussion, you could add the data on Swedish patients regarding the time intervals (even though no big discrepancies seem to be evident).

*Response: The reference has been added.*

The strengths and limitations of this study are well discussed.

Tables and figures seem to be appropriate and their headings, too.

Journal requirements:

When submitting your revision, we need you to address these additional requirements.

1.  Please ensure that your manuscript meets PLOS ONE's style requirements, including those for file naming. The PLOS ONE style templates can be found at

[https://journals.plos.org/plosone/s/file?id=wjVg/PLOSOne_formatting_sample_main_body.pdf](https://eur03.safelinks.protection.outlook.com/?url=https%3A%2F%2Fjournals.plos.org%2Fplosone%2Fs%2Ffile%3Fid%3DwjVg%2FPLOSOne_formatting_sample_main_body.pdf&data=05%7C01%7C%7C69b115cae30c483c732408db06005513%7C5968b90c51a64f088b4750ffffbe2e4f%7C0%7C0%7C638110370765139665%7CUnknown%7CTWFpbGZsb3d8eyJWIjoiMC4wLjAwMDAiLCJQIjoiV2luMzIiLCJBTiI6Ik1haWwiLCJXVCI6Mn0%3D%7C3000%7C%7C%7C&sdata=MlB7UIh%2Fnj9T3YXyw7Q9UQd12nRbqomfxcYX659fzGs%3D&reserved=0)  and

[https://journals.plos.org/plosone/s/file?id=ba62/PLOSOne_formatting_sample_title_authors_affiliations.pdf](https://eur03.safelinks.protection.outlook.com/?url=https%3A%2F%2Fjournals.plos.org%2Fplosone%2Fs%2Ffile%3Fid%3Dba62%2FPLOSOne_formatting_sample_title_authors_affiliations.pdf&data=05%7C01%7C%7C69b115cae30c483c732408db06005513%7C5968b90c51a64f088b4750ffffbe2e4f%7C0%7C0%7C638110370765295909%7CUnknown%7CTWFpbGZsb3d8eyJWIjoiMC4wLjAwMDAiLCJQIjoiV2luMzIiLCJBTiI6Ik1haWwiLCJXVCI6Mn0%3D%7C3000%7C%7C%7C&sdata=0ldWbIcDHCLO0%2Bz8VjYE3WBIihkjMy7Y9sHdbh%2B%2BVYA%3D&reserved=0)

*Response: The headings has been altered. The figure titles has been made bold. The corresponding author line has been adjusted. Acknowledgement has been moved to after discussion and conclusion*

2. Please ensure that you have specified (1) whether consent was informed and (2) what type you obtained (for instance, written or verbal, and if verbal, how it was documented and witnessed). If your study included minors, state whether you obtained consent from parents or guardians. If the need for consent was waived by the ethics committee, please include this information.

*Response: a sentence declaring that formal consent is not required for the study has been added in the ethics section.*

3. We note that the grant information you provided in the ‘Funding Information’ and ‘Financial Disclosure’ sections do not match.

When you resubmit, please ensure that you provide the correct grant numbers for the awards you received for your study in the ‘Funding Information’ section.

*Response: the financial disclosure section has been altered to match the section of funding information.*

4. We note that you have stated that you will provide repository information for your data at acceptance. Should your manuscript be accepted for publication, we will hold it until you provide the relevant accession numbers or DOIs necessary to access your data. If you wish to make changes to your Data Availability statement, please describe these changes in your cover letter and we will update your Data Availability statement to reflect the information you provide.

5. We note that you have included the phrase “data not shown” in your manuscript. Unfortunately, this does not meet our data sharing requirements. PLOS does not permit references to inaccessible data. We require that authors provide all relevant data within the paper, Supporting Information files, or in an acceptable, public repository. Please add a citation to support this phrase or upload the data that corresponds with these findings to a stable repository (such as Figshare or Dryad) and provide and URLs, DOIs, or accession numbers that may be used to access these data. Or, if the data are not a core part of the research being presented in your study, we ask that you remove the phrase that refers to these data.

*Response: the sentence has been removed since the data is accessible.*
